# Physicochemical and Fibril Formation Properties of Pufferfish (*Takifugu obscurus*) Skin Collagen from Solvent Extraction in Different Conditions

**DOI:** 10.3390/gels9010017

**Published:** 2022-12-26

**Authors:** Shanshan Wang, Deqing Zhou, Nan Liu, Yong Sun, Guohui Sun

**Affiliations:** 1 Yellow Sea Fisheries Research Institute, Chinese Academy of Fishery Sciences, Qingdao 266071, China; 2 Laboratory for Marine Drugs and Bioproducts, Pilot National Laboratory for Marine Science and Technology (Qingdao), Qingdao 266237, China

**Keywords:** pufferfish skin, acid-solubilized collagen, pepsin-solubilized collagen, fibril formation, microstructural characteristic

## Abstract

Acid-solubilized (ASC) and pepsin-solubilized collagen (PSC) extracted at 4 °C (ASC-4 and PSC-4), 12 °C (ASC-12 and PSC-12), and 20 °C (ASC-20 and PSC-20) from the skin of farmed pufferfish (*Takifugu obscurus*) was characterized by SDS-polyacrylamide gel electrophoresis (SDS-PAGE), Fourier-transform infrared spectroscopy (FTIR), and fibril-forming tests. The results indicate that extraction at 12 °C can effectively improve the extraction efficiency of natural collagen compared with extraction at 4 °C. However, extraction at 20 °C results in a decrease in molecular integrity, thus, inducing the resultant collagen to degrade or even lose fibril-forming ability. Transmission electron microscope (TEM) images revealed that ASC-4, PSC-4, ASC-12, and PSC-12 can assemble into fibrils with D-periodicities, and ASC-20 associated into molecular aggregates alongside partial D-banded fibrils, while no well-defined fibrils were observed in PSC-20. Scanning electron microscope (SEM) analysis confirmed the well-defined fibril morphologies of ASC-4, PSC-4, ASC-12, and PSC-12 with imino acid contents between 190.0 and 197.8 residues/1000 residues. The denaturation temperature of ASC-4, PSC-4, ASC-12 and PSC-12 was 30.0, 27.6, 25.9 and 22.7 °C, respectively. This study indicates that ASC and PSC extracted at 4 °C and 12 °C could be alternatives to terrestrial collagens for industrial applications.

## 1. Introduction

Collagen is the most abundant extracellular protein found in skin, bone, tendon, ligament, and corneal and other connective tissues [1]. The hierarchical structure of collagen, formed from tropocollagen monomers into micrometer-scale fibers, is the foundation behind the mechanical strength of the collagen found in almost all load-bearing tissues [2,3]. Due to the advantages of high biocompatibility, biodegradability, and low antigenicity, collagen-based polymer materials have been rapidly developed for wound dressings or bioengineered scaffolds in the pharmaceutical fields and packaging films for meat products or sausage casing in the food industry [4,5]. Nowadays, industrial collagens are mainly manufactured from bovine, porcine, and poultry skins and bones. However, food safety crises and sociocultural reasons have resulted in a demand for collagen from alternative sources, especially from aquatic organisms [6,7,8,9].

Pufferfish (*Takifugu obscurus*), a fish species of economic importance, belongs to the family Tetradontidae of teleost fish. Pufferfish are famous for their puffing behavior and have become increasingly popular among consumers for their non-toxicity, desirable flavor, and high nutritional value [10]. However, the consumption of pufferfish was banned in mainland China for 26 years due to the potential existence of tetrodotoxin in wild species. In 2016, the consumption of farmed pufferfish was conditionally approved by the Ministry of Agriculture, the National Health and Family Planning Commission, and the China Food and Drug Administration. Thus, the production volume of farmed pufferfish is expected to increase. In 2021, the freshwater production of pufferfish was 14,559 t; therefore, the efficient utilization of processing byproducts, including fish skins, has become an important task [11].

So far, skin collagen has been extracted from many fish species, including barramundi (*Lates calcarifer*) [12], lizardfish (*Saurida tumbil*) [13], parrotfish (*Chlorurus sordidus*) [14], sturgeon (*Huso huso*) [15], Greenland halibut (*Reinhardtius hippoglossoides*) [16] and rainbow trout (*Oncorhynchus mykiss*) [17]. Traditionally, low-concentration, acid-aided extraction at a low temperature (usually 4 °C) has been the most common method to extract collagen from natural tissues [18]. However, low-temperature extraction is a time-consuming process and generates substantial undissolved residues. Previous studies reported that the recovery rate and extraction efficiency could be increased by using pepsin or extracting at a higher temperature [19,20]. Limited pepsin digestion could cleave the cross-linked collagen molecules at telopeptide regions without impairing the triple-helical structure, thus, increasing the recovery rate and further removing the antigenic P determinant located on the non-helical sections [21]. Optimum extraction temperature could improve the extraction efficiency. However, an increase in temperature, to a certain extent, may also denature the collagen and change the physicochemical properties [22]. Thus, the objective of this study was to improve the extraction efficiency while preserving the structural integrity of collagen from pufferfish skins. The effect of pepsin treatment and extraction temperature (4, 12, and 20 °C) on the extraction efficiency and physicochemical characteristics, including fibril formation abilities, of resultant collagens was determined.

## 2. Results and Discussion

### 2.1. Proximate Analysis

The proximate composition of pufferfish skin is moisture (72.15 ± 2.55%), ash (2.84 ± 0.11%), protein (24.12 ± 0.86%), and fat (0.51 ± 0.05%). The protein content in pufferfish skin is higher than that of Spanish mackerel (17.17%) and Nile perch (21.6%) [6,23]. In addition, the crude protein content on a dry basis is 86.61%, which is similar to that of pufferfish (*Takifugu rubripes*) (89.31%) [24]. Relatively higher content of protein makes pufferfish skin a good source for aquatic collagen extraction.

### 2.2. Recovery Rate

**Figure 1:**
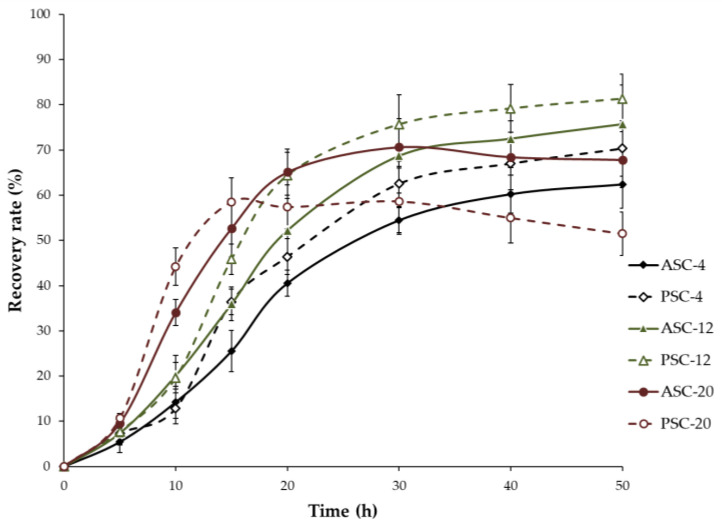
Recovery rate (%) of ASC and PSC extracted at 4, 12 and 20 °C from the skin of pufferfish.

The recovery rate of extracted collagen of all treatments is shown in Figure 1. With extraction at low temperature, the recovery rate of PSC-4 (62.57%) was higher than that of ASC-4 (54.44%) after 30 h of extraction (*p* < 0.05), indicating that pepsin treatment can efficiently cleave the cross-linked collagen molecules at telopeptide regions and induce extraction with higher yield. Extraction temperature also had significant influence on the extraction efficiency. The recovery rate of ASC-12 (52.21%) and PSC-12 (63.41%) was markedly increased when compared with that of ASC-4 (40.52%) and PSC-4 (46.42%) after 20 h of extraction (*p* < 0.05). However, with extraction at a higher temperature (20 °C), the recovery rate of PSC-20 (57.42%) was lower than that of ASC-20 (65.11%) after 20 h of extraction (*p* < 0.05) and then decreased continuously as extraction time proceeded. This result might be attributed to the excessive pepsin digestion at the high temperature, resulting in an increase in lower-MW (molecular weight) protein fragments. Thus, pepsin treatment and extraction temperature are two important parameters that influence the recovery rate and extraction efficiency. Samples acquired under the condition that produced a high recovery rate at each extraction temperature (ASC-4, PSC-4, ASC-12, and PSC-12 for 50 h, ASC-20 and PSC-20 for 30 h) were prepared for the subsequent experiments.

### 2.3. Sodium Dodecyl Sulfate Polyacrylamide Gel Electrophoresis (SDS-PAGE)

**Figure 2:**
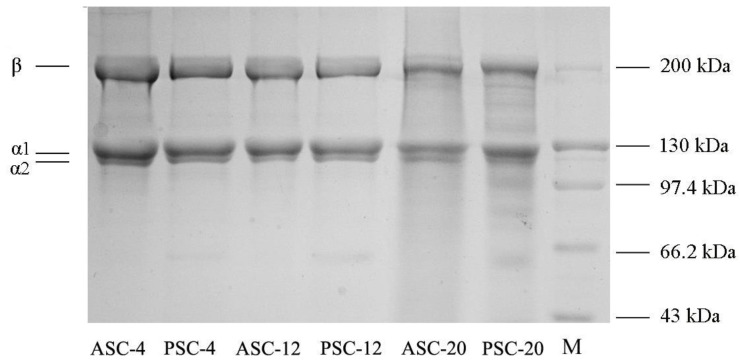
SDS-PAGE patterns of ASC and PSC extracted at 4, 12 and 20 °C from the skin of pufferfish. ASC: acid-solubilized collagen; PSC: pepsin-solubilized collagen; M: protein marker.

The SDS-PAGE analysis showed that all extracted samples were composed of one β chain and at least two α chains (α1 and α2) as their major subunits (Figure 2). The molecule weights of the α1 and β chain were approximately 130 and 200 kDa, respectively. The subunit composition of all samples was similar to that of type I collagen from other fish species [17,25], indicating that collagen from pufferfish *T. obscurus* skin might be type I collagen. The subunit composition of ASC-4, PSC-4, ASC-12, PSC-12 and ASC-20 was similar, suggesting that the extraction temperature applied does not significantly induce collagen molecule chains to degrade. However, PSC-20 contained reduced proportions of β-chain as well as low-MW fragments (<97.4 kDa), indicating that high-Mw chains are partially degraded by excessive pepsin digestion at higher temperature.

### 2.4. Fourier-Transform Infrared Spectroscopy (FTIR)

**Figure 3:**
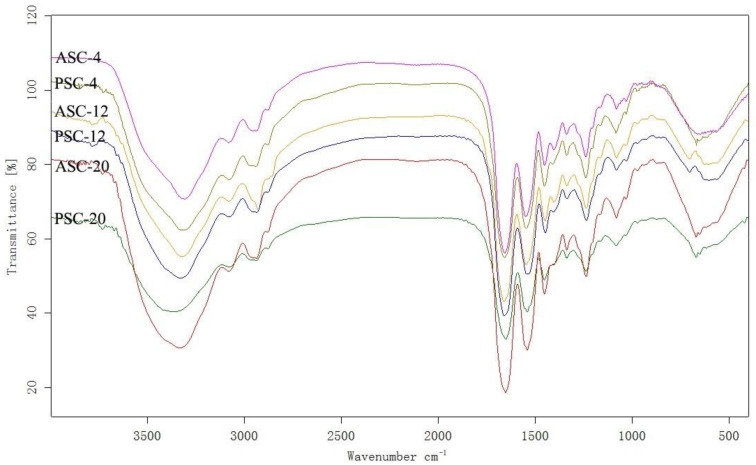
FTIR spectra of ASC and PSC extracted at 4, 12 and 20 °C from the skin of pufferfish. ASC: acid-solubilized collagen; PSC: pepsin-solubilized collagen.

FTIR spectra exhibited four amides (amide A, I, II and III) identified as the major peaks of extracted collagens (Figure 3). Amide A (3400–3440 cm^−1^) is related to N–H stretch coupled with hydrogen bonds and moves to a lower frequency (usually near 3300 cm^−1^) when N–H groups are involved in the formation of hydrogen bonds [26]. The amide A bands of ASC-4, PSC-4, ASC-12, PSC-12, ASC-20 and PSC-20 were found at 3307, 3308, 3323, 3322, 3335 and 3359 cm^−1^, respectively. This result indicates that fewer N–H groups in collagen extracted at higher temperature are involved in hydrogen bond formation, suggesting the triple-helical structure is less intact.

The amide I band is associated with the stretching vibrations of the carbonyl group (C=O) or hydrogen bond coupled with COO^−^ and is the important factor in investigating the secondary structure of proteins [27]. The amide II band is caused by N–H bending coupled with C–N stretching. A shift of amide I and II bands to lower wavenumbers is associated with a decrease in molecular order [28,29]. The amide I bands of ASC-4, PSC-4, ASC-12, PSC-12, ASC-20 and PSC-20 were observed at 1658, 1659, 1660, 1659, 1653 and 1652 cm^−1^, respectively; meanwhile, the amide II bands were detected at 1550, 1548, 1548, 1545, 1542 and 1541 cm^−1^, respectively. This result represents that the degree of molecular order of ASC-20 and PSC-20 is relatively lower than that of ASC-4, PSC-4, ASC-12 and PSC-12. The amide III band is associated with C–N stretching and N–H deformation from amide linkages as well as the absorption caused by the wagging vibration of the CH_2_ groups of the glycine backbone and the proline side chains [30]. The amine III bands were confirmed in the range of 1236–1240 cm^−1^. Based on SDA-PAGE and FTIR spectra data, extraction at higher temperature with pepsin digestion results in a decrease in molecule order in extracted collagen.

### 2.5. In Vitro Fibril Formation Ability

In vivo, collagen molecules can undergo self-assembly into highly ordered supramolecular structures and further intertwine into fibrils which can form even larger bundles [31]. Previous studies have shown that in-vitro-purified collagen can also form fibrils with the same axial periodicity as native fibrils under suitable conditions [32]. The critical structure factor of the self-assembly process is the alternation of charged and hydrophobic side chains on the helix surface, which is determined by amino acid sequences [33]. Aquatic collagen with a strong fibril formation property can be regarded as an excellent alternative to the collagen of land-based animals.

**Figure 4:**
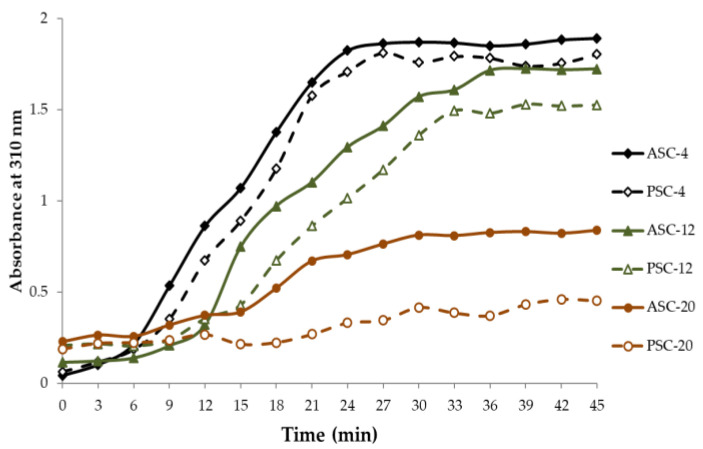
Turbidity–time curves of resulting fibrils of ASC and PSC extracted at 4, 12 and 20 °C from the skin of pufferfish. ASC: acid-solubilized collagen; PSC: pepsin-solubilized collagen.

As shown in Figure 4, ASC-4, PSC-4, ASC-12, PSC-12 and ASC-20 exhibited typical sigmoidal turbidity–time curves with an initial lag phase followed by a growth phase and then a plateau phase. However, the absorbance at 310 nm of ASC-20 was lower than that of ASC-4, PSC-4, ASC-12 and PSC-12. Meanwhile, the turbidity–time curve of PSC-20 was almost straight, revealing that PSC-20 might not be able to effectively form fibrils. The fibril formation degree of extracted collagen was 94.42% (ASC-4), 89.62% (PSC-4), 84.36% (ASC-12), 72.80% (PSC-12), 48.31% (ASC-20) and 27.14% (PSC-20), respectively. It has been reported that fibril formation ability is related to molecular integrity [19].

Therefore, it was speculated that higher extraction temperature with pepsin treatment could impair the structural integrity in whole or in part, thus, causing a decrease in fibril formation ability. It can also be seen in Figure 4 that the fibril formation rate of PSCs (PSC-4 and PSC-12) was relatively slower than that of their corresponding ASCs (ASC-4 and ASC-12), indicating that the nucleation time of collagen fibrils is prolonged for pepsin hydrolysis. Telopeptide at the N- and C-termini of the triple-helical domains is important in stabilizing initial aggregates. Previous studies reported that acid-solubilized collagen with preserved telopeptide regions can easily undergo self-assembly at optimal conditions. Thus, the initiation process of enzymatic-pretreated collagen is delayed compared with that of the intact collagen, and the resultant fibrillary lattice might exhibit a lower density and larger diameters of pores [34].

### 2.6. Transmission Electron Micrographs (TEM)

**Figure 5:**
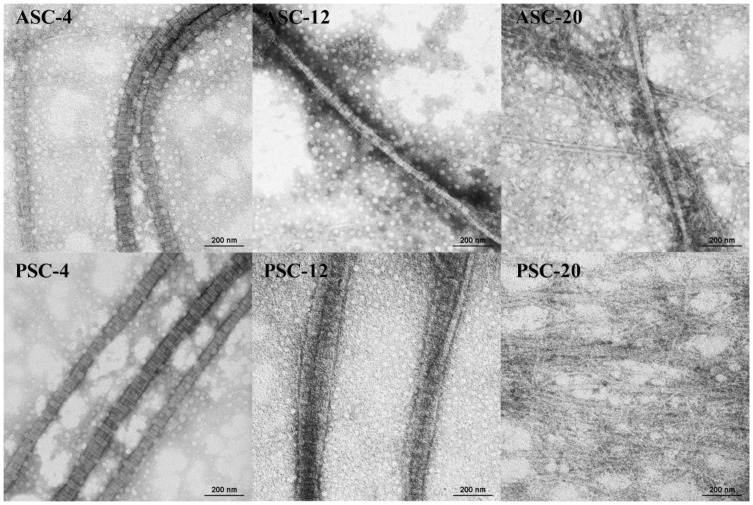
TEM images of resulting fibrils of ASC and PSC extracted at 4, 12 and 20 °C from the skin of pufferfish. ASC: acid-solubilized collagen; PSC: pepsin-solubilized collagen. Scale bars, 200 nm.

The TEM images of the resulting fibrils are shown in Figure 5. ASC-4, PSC-4, ASC-12 and PSC-12 could form parallel-sided fibrils with characteristic D-periodicity similar to that observed in rat tail collagen [35]. It was found that ASC-20 molecules have a tendency to associate into disorganized molecular aggregates alongside partial D-banded fibrils. For PSC-20, no well-banded fibrils were observed within the poorly aligned molecular aggregates.

D-periodical banding serves as an indication for the reconstruction of native-like fibrils and is important in maintaining the mechanical stability and biological functions of tissues [36]. It has been reported that D-periodicity is an attribute of highly ordered structure which settles surface properties and nanomechanical characteristics. D-periodicity also results in the regular organization of collagen-binding sites on the fibril surfaces [34]. A strong correlation of cell elongation and motion directionality with the orientation of D-periodic collagen fibrils was observed, whereas neither directed motility nor cell body alignment was found on aligned collagen without D-periodicity [37]. Some ultrastructural studies showed conformational changes in collagen fibrils as a result of deformation [38].

Combined with the turbidity–time curves and fibril-forming degree, the results revealed that pepsin treatment influences the fibril-forming rate of the extracted collagens, and collagen lacking telopeptides might form a looser fibrillary structure [32,39], while higher extraction temperature can significantly induce collagen to reduce or even lose fibril-forming ability. Thus, collagen extracted at 4 °C and 12 °C has the ability to be utilized as a novel source for biomaterials and, therefore, was prepared for subsequent experiments.

### 2.7. Scanning Electron Micrographs (SEM)

**Figure 6:**
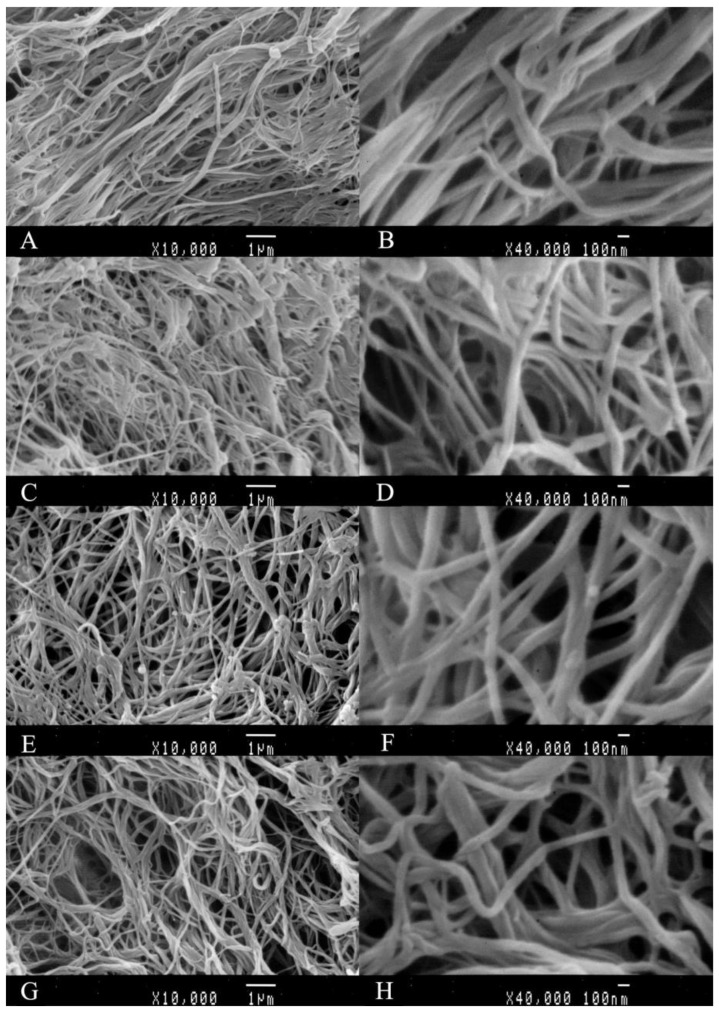
SEM images of resulting fibrils of ASC and PSC extracted at 4 and 12 °C from the skin of pufferfish. (**A**) ASC-4 (×10,000); (**B**) ASC-4 (×40,000); (**C**) PSC-4 (×10,000); (**D**) PSC-4 (×40,000); (**E**) ASC-12 (×10,000); (**F**) ASC-12 (×40,000); (**G**) PSC-12 (×10,000); (**H**) PSC-12 (×40,000). Scale bars, 1 μm (**A**,**C**,**E**,**G**); 100 nm (**B**,**D**,**F**,**H**).

The SEM images of resulting fibrils are shown in Figure 6. The fibrils of ASC-4, PSC-4, ASC-12 and PSC-12 were oriented in various directions and entangled with each other. The diameter of the resulting fibrils was around 100 nm, which is similar to the native fibrils formed in vivo [40]. Thick fibril bundles with diameters larger than 100 nm were also observed, which were formed by the laterally associated fibril monomers. Previous studies reported that collagen fibrils found in tissues can vary in diameter from 80 nm to over 400 nm [28]. Similar fibril morphologies were observed in collagen from the skin of Bester sturgeon and grass carp [25,41]; however, the ultrastructure of resulting fibrils is different to that of fibrils from catla skin collagen, which has nodular-like structures [42]. The differences in morphological characteristics might be explained by the differences in the species and conformations of collagens [43]. In view of the increasing applications of collagen-based materials, collagen extracted at 4 and 12 °C from the skin of pufferfish has the potential to serve as tissue engineering scaffolds, 3D cell culture systems, and food packaging films.

### 2.8. Amino Acid Composition

Amino acid compositions of four extracted collagens expressed as residues per 1000 total amino acid residues are shown in Table 1. Glycine was the major amino acid, followed by alanine, proline, and hydroxyproline. The amino acids were divided into three groups: hydrophobic amino acids (649.9–660.3 residues/1000 residues), charged amino acids (185.0–192.5 residues/1000 residues), and polar amino acids (65.7–67.3 residues/1000 residues). All four samples were rich in hydrophobic amino acids, which were higher than that of *Loligo vulgaris* squid mantle collagen (585–591 residues/1000 residues) [21]. Hydrophobic amino acids can upgrade the antioxidant as well as ACE-inhibiting activities of collagen-derived hydrolysates, since hydrophobic amino acids facilitate the protein molecules to access the hydrophobic targets and, therefore, increase the affinity and reactivity [44]. This result indicates that collagen extracted from farmed pufferfish skin can be utilized as a potential source to produce antioxidant and antihypertensive peptides.

The imino acid (proline + hydroxyproline) contents of ASC-4, PSC-4, ASC-12 and PSC-12 were 192.6, 197.8, 191.6, and 190.0 residues/1000 residues, which are similar to those of unicorn leatherjacket (187–190 residues/1000 residues) [45], higher than those of cold-water species such as cod (154 residues/1000 residues) [46], and lower than calf and pig skin collagen (216.6–220.0 residues/1000 residues) [47]. Imino acid content is highly correlated with the different environmental and body temperatures of their original organisms. A lower content of imino acid can lead to inferior thermal stability and a lower melting point [48].

### 2.9. Determination of Denaturation Temperature (T_d_)

**Figure 7:**
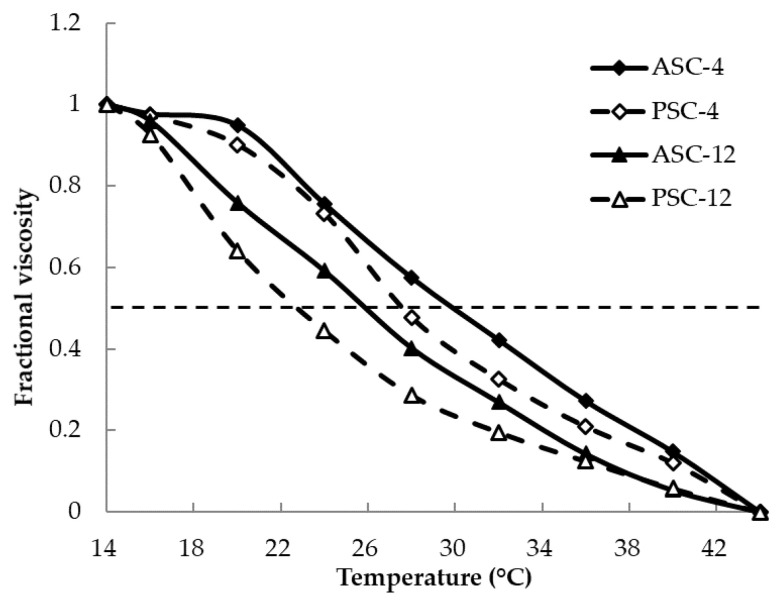
Thermal denaturation curve of ASC and PSC extracted at 4 and 12 °C from the skin of pufferfish.

The *T_d_* values of ASC-4, PSC-4, ASC-12 and PSC-12 were 30.0, 27.6, 25.9 and 22.7 °C, respectively (Figure 7). Generally, *T_d_* value is influenced by the imino acid content, which contributes to the structural integrity of collagen. However, the imino acid contents of ASC-4, PSC-4, ASC-12 and PSC-12 were similar (Table 1). These changes could be caused by the telopeptide reduction and the loss of molecular integrity by pepsin digestion at a high temperature.

The *T_d_* value of ASC-4 and PSC-4 (27.6–30.0 °C) was higher than that of collagens from the skin of Spanish mackerel (14.7–15.1 °C) and rainbow trout (21.4–21.5 °C) [6,17], but lower than that of calf skin collage (37 °C) [29]. This result is in agreement with previous reports that the thermal stability of collagen extracted from an organism is correlated with its body and habitat temperatures [49]. Moreover, the *T_d_* of ASC-4 and PSC-4 was higher than that of several invertebrates, including *Cyanea nozakii* jellyfish (23.8 °C) and *Loligo vulgaris* squid (21–22 °C) [21,50]. Therefore, collagen from pufferfish skin might be used as a better source for collagen-based materials than the collagen of cold-water fish and invertebrate species.

### 2.10. Solubility

**Figure 8:**
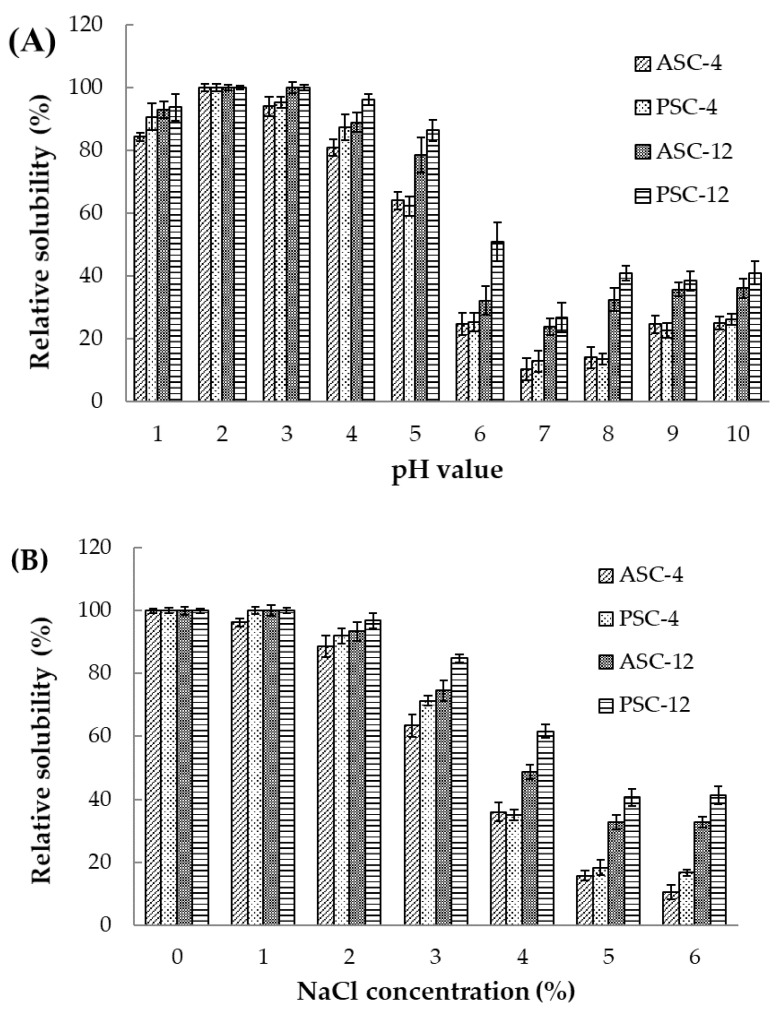
The effect of pH (**A**) and NaCl (**B**) concentration on the solubility of ASC and PSC extracted at 4 and 12 °C from the skin of pufferfish.

The effect of pH on the solubility of extracted collagens is shown in Figure 8A. All the samples exhibited high solubility in acid conditions at a pH between 2 and 3. A sharp decrease in solubility was observed for all four samples in the pH range of 4–7. The solubility was then slightly increased after reaching a minimum at pH 7.0. The aggregation and precipitation of collagen were induced by the almost zero net charge and the enhancing hydrophobic–hydrophobic interaction at its pI. Therefore, the isoelectric point (pI) for collagens extracted from pufferfish skin was obtained at pH values around 7, which are similar to the results of previous studies where collagen pI values ranged from pH 6 to 9 [21].

The solubility values of extracted collagens decreased sharply at a high NaCl concentration above 2% (Figure 8B). The precipitation of collagen might be caused by the salting-out effect induced by the hydrophobic–hydrophobic interactions between molecular chains with increasing ionic strength [6]. In addition, at the same pH point and NaCl concentration, ASC-12 and PSC-12 exhibited a higher solubility than ASC-4 and PSC-4, which might be explained by the higher degree of triple-helical structure preserved in collagen extracted at 4 °C. Moreover, the PSCs (PSC-4 and PSC-12) displayed a relatively higher solubility than their corresponding ASCs (ASC-4 and ASC-12), which might be due to the partial hydrolysis of the high-MW, cross-linked components in PSCs. The solubility characteristics of extracted collagens with changes in pH and NaCl concentrations may be important in their further applications.

## 3. Conclusions

Acid-solubilized and pepsin-solubilized collagen (type I) was prepared from the skin of pufferfish at different temperatures. Results showed that an extraction temperature of 12 °C markedly increases the yield and extraction efficiency compared to an extraction temperature of 4 °C. However, extraction at 20 °C results in a degradation of subunit integrity, which leads to the formation of poorly banded and not well-ordered fibrillary aggregates. Moreover, the triple-helical structure of collagens extracted at 4 °C and 12 °C is maintained, as observed under FTIR spectra, turbidity assay, and TEM analyses. In conclusion, ASC-4, PSC-4, ASC-12 and PSC-12 exhibit better fibril formation abilities and have the potential to be explored further as an alternative collagen source.

## 4. Materials and Methods

### 4.1. Chemicals

Acetic acid, sodium chloride, sodium dodecyl sulfate (SDS), ammonium persulfate, and Coomassie Brilliant Blue R-250 were purchased from Sinopharm Chemical Reagent Co., Ltd. (Beijing, China). High-MW marker was purchased from Solarbio Biotechnology Co., Ltd. (Beijing, China). Pepsin (EC 3.4.23.1) was purchased from Sigma-Aldrich Chemical Company (St. Louis, MO, USA). All other reagents used were of analytical grade.

### 4.2. Proximate Analysis

Moisture, ash, crude protein, and fat of raw materials were measured according to the procedures of AOAC (2003) [51] method no. 950.46B, 920.153, 981.10, 960.39 (a).

### 4.3. Treatment of Fish Skin

Pufferfish (*T. obscurus*) skin was obtained from Shenshi Aquatic Product Co., Ltd. (Taizhou, China). Fresh skin was kept in ice at a ratio of 1:2 (*w*/*w*) and transported to the laboratory within 24 h. Upon arrival, fish skin was washed with iced tap water (0–4 °C) and cut into small pieces (approximately 0.5 × 0.5 cm^2^). To remove non-collagenous protein and pigment, skin pieces were soaked in 0.1 M NaOH with a skin/solution ratio of 1:20 (*w*/*v*) for 12 h. The mixture was continuously stirred, and the alkaline solution was changed every 4 h. Alkali-treated skin was washed with distilled water until pH was neutral, homogenized, and then lyophilized [6].

### 4.4. Extraction of Acid-Solubilized Collagen (ASC)

Pretreated skin was soaked in 0.5 M acetic acid with a skin/solvent ratio of 1:50 (*w*/*v*). Extraction procedures were conducted at different temperatures (4, 12 and 20 °C) for 0–50 h with continuous stirring following the method of Li et al. [6] and Shen et al. [41] with slight modifications. The mixture was centrifuged at 10,000× *g* for 30 min at 4 °C. Then, ASC in the supernatant was precipitated by adding NaCl to a final concentration of 0.9 M and collected by centrifuging at 10,000× *g* for 30 min at 4 °C. The precipitates were re-dissolved in a minimum volume of 0.5 M acetic acid, dialyzed at 4 °C against 0.1 M acetic acid for 12 h and distilled water for 48 h, and then lyophilized. The obtained ASCs extracted at 4, 12 and 20 °C were referred to as ASC-4, ASC-12 and ASC-20, respectively.

### 4.5. Extraction of Pepsin-Solubilized Collagen (PSC)

Pretreated skin was soaked with 0.5 M acetic acid in a ratio of 1:50 (*w*/*v*) containing 1% (*w*/*w*) pepsin. Extraction procedures were conducted at different temperatures (4, 12, and 20 °C) for 0–50 h with continuous stirring following the method of Li et al. [6] and Shen et al. [41] with slight modifications. The PSC in the supernatant was salted out by addition of NaCl to 0.9 M and collected by centrifuging at 10,000× *g* for 30 min at 4 °C. Precipitate collagen was re-dissolved in 0.5 M acetic acid, dialyzed against 0.02 M Na_2_HPO_4_ solution for 12 h, 0.1 M acetic acid for 12 h, and distilled water for 48 h, and then lyophilized. The obtained PSCs extracted at 4, 12 and 20 °C were referred to as PSC-4, PSC-12 and PSC-20, respectively.

### 4.6. Recovery Rate

The hydroxyproline content in fish skin and extracted collagen was determined according to the method of Reddy and Enwemeka [52]. The recovery rate (%) was calculated as the ratio of hydroxyproline content in extracted collagen to hydroxyproline content in fish skin.

### 4.7. SDS-PAGE

SDS-PAGE was performed following the procedure of Laemmli [53] with slight modifications. Collagen samples were dissolved in loading buffer (60 mM Tris-HCl, pH 8.0, containing 25% glycerol, 2% SDS and 0.1% bromophenol blue) in the presence of β-mercaptoethanol and then loaded onto a polyacrylamide gel made of 7.0% running gel and 4.0% stacking gel.

### 4.8. FTIR Analysis

FTIR spectra were measured from discs containing 2 mg samples in approximately 100 mg spectrum-pure KBr ground together under drying conditions according to the method of Cozza et al. [21]. The spectra were recorded using an infrared spectrophotometer (200SXV, Nicolet, Madison, WI, USA) at a data acquisition rate of 2 cm^−1^ per point.

### 4.9. Fibril Formation In Vitro

Fibril formation ability was measured following the method of Pal et al. [42] with slight modifications. Samples were dissolved in 0.5 M acetic acid to 0.3% (*w*/*v*) concentration at 4 °C. The obtained solution was mixed with 0.1 M phosphate buffer (pH 7.4), and the final pH was adjusted to neutral (pH 7.0 ± 0.2). The solution was kept at 25 °C to reconstruct the fibrils. Fibril formation rate was obtained by measuring the turbidity changes by the increase in absorbance at 310 nm using a UV–visible spectrophotometer (UV-2550, Shimadzu Ltd., Tokyo, Japan). After the fibril formation experiment, the mixture was centrifuged at 8000× *g* for 30 min. Fibril formation degree was calculated as follows:

(1) $$\mathrm{Fibril}\;\mathrm{formation}\;\mathrm{degree}\;(\%)=(1-\frac{Hydroxyproline\;content\;in\;the\;supernatant}{Hydroxyproline\;content\;in\;the\;collagen\;sample})\;\times \;100$$

### 4.10. TEM Observation

The TEM observations of collagen fibrils were obtained following the method of Li and Douglas [36]. Samples were prepared by placing the fibril suspensions on 200 mesh copper grids and staining them with 1% (*w*/*v*) phosphotungstic acid. TEM images were observed using a transmission electron microscope (JSM-1200, JEOL Ltd., Tokyo, Japan).

### 4.11. SEM Observation

The SEM observations of fibril samples were obtained according to the method of Zhang et al. [25] with slight modifications. Fibril samples were fixed with 2.5% (*v*/*v*) glutaraldehyde for 12 h, then rinsed with 0.1 M phosphate buffer (pH 7.2). After dehydration in a graded series of ethanol, fibril samples were dried in a critical point dryer (HCP-2, Hitachi Ltd., Tokyo, Japan). A scanning electron microscope (JSM-840, JEOL Ltd., Tokyo, Japan) was applied to observe the SEM morphology at 10,000× and 40,000× magnification.

### 4.12. Amino Acid Composition

Amino acid compositions were analyzed following the method of Li et al. [6]. Collagen samples were hydrolyzed in 6 M HCl at 110 °C under vacuum for 24 h and then evaporated. The remaining residue was mixed with 25 mL citric acid buffer and then applied to the automated amino acid analyzer (835-50, Hitachi Ltd., Tokyo, Japan).

### 4.13. Determination of Denaturation Temperature

The denaturation temperatures (*T_d_*) were determined following the method of Yan et al. [54] with slight modifications. Thermal determination curves were obtained by measuring the viscosity from 14 to 44 °C using a viscometer (MCR101, Anton Paar Ltd., Shanghai, China). Fractional viscosity was calculated as:

Fractional viscosity = (η_sp(measured)_ − η_sp(minimum)_)/(η_sp(maximum)_ − η_sp(minimum)_), where η_sp_ is the specific viscosity. *T_d_* was determined as the temperature at which the change in viscosity was 50% decreased.

### 4.14. Solubility

The solubility of extracted collagens, as influenced by pH and NaCl concentration, was determined by the methods of Shen et al. [41]. Samples were dissolved in 0.5 M acetic acid at 4 °C for 12 h to a concentration of 3 mg/mL. The pH of 8 mL sample solutions was adjusted from 1 to 10. The total volume of sample solution was taken up to 10 mL with 0.5 M acetic acid previous adjusted to the same pH as required. The mixtures were stirred for 20 min at 4 °C, then centrifuged at 10,000× *g* for 30 min to remove undissolved debris. Protein content in the supernatant was determined using Bradford protein assay (Solarbio Ltd., Beijing, China). The relative solubility was calculated as a percentage of that obtained at the pH giving the highest solubility.

Samples were redissolved in 0.5 M acetic acid at 4 °C for 12 h to a concentration of 6 mg/mL. An 8 mL amount of sample solution was mixed with an equal volume of NaCl in 0.5 M acetic acid solution to obtain final concentrations of 1, 2, 3, 4, 5 and 6%. The mixtures were stirred for 20 min at 4 °C, then centrifuged at 10,000× *g* for 30 min. Protein content in the supernatant was determined as described above. The relative solubility was calculated in comparison to the total collagen in 0.5 M acetic acid.

### 4.15. Statistical Analysis

An analysis of variance (ANOVA), followed by Duncan’s multiple-range test, was used for comparisons by SPSS 25.0 software (SPSS Inc., Chicago, IL, USA). Experiments were performed in triplicate. Mean values with standard deviations (SD) were reported. Difference was considered to be significant if *p* < 0.05.

## Figures and Tables

**Table 1:** Amino acid composition of ASC and PSC extracted at 4 and 12 °C from the skin of pufferfish (residues/1000 residues).

Amino Acid	ASC-4	PSC-4	ASC-12	PSC-12
Aspartic acid (Asp)	43.2	45.5	42.0	48.9
Threonine (Thr)	22.6	18.5	21.2	17.1
Serine (Ser)	33.4	37.0	32.8	38.6
Glutamic acid (Glu)	74	69.5	72.7	73.6
Glycine (Gly)	351.9	344.6	351.1	355.3
Alanine (Ala)	115.8	121.0	118.4	115.5
Cysteine (Cys)	0.8	0.9	1.4	1.1
Valine (Val)	21.7	23.8	22.3	20.1
Methionine (Met)	10.5	9.7	11.9	9.1
Isoleucine (Ile)	10.9	10.8	11.6	8.9
Leucine (Leu)	19.5	16.6	18.1	20.1
Tyrosine (Tyr)	3.6	4.0	4.7	5.2
Phenylalanine (Phe)	11.3	12.4	16.1	14.4
Histidine (His)	6.1	6.9	5.6	4.9
Lysine (Lys)	23.5	25.9	22.4	24.2
Arginine (Arg)	49.4	47.7	47.9	45.8
Proline (Pro)	113.5	116.3	110.8	106.5
Hydroxyproline (Hyp)	79.1	81.5	80.8	83.5
Hydroxylysine (Hyl)	9.2	7.4	8.2	7.2
Total	1000	1000	1000	1000
THAA ^1^	655.1	655.2	660.3	649.9
TCAA ^2^	190.1	188.6	185.0	192.5
TPAA ^3^	66.5	67.3	65.7	66.9
Imino acid ^4^	192.6	197.8	191.6	190.0

^1^ THAA (total hydrophobic amino acids): ∑ proline + alanine + valine + methionine + glycine + isoleucine + leucine + phenylalanine. ^2^ TCAA (total charged amino acids): ∑ aspartic acid + glutamic acid + arginine + lysine. ^3^ TPAA (total polar amino acids): ∑ serine + histidine + threonine + cysteine + tyrosine. ^4^ Imino acid: proline + hydroxyproline.

## Data Availability

Not applicable.
